# Three‐dimensional full‐scale bone modeling for preoperative simulation of surgery in patients with elbow contractures due to bone deformities

**DOI:** 10.1002/ccr3.1003

**Published:** 2017-07-15

**Authors:** Naoshige Nagura, Tomonori Kenmoku, Kenji Onuma, Kensuke Fukushima, Hisako Fujimaki, Naonobu Takahira, Masashi Takaso

**Affiliations:** ^1^ Department of Orthopaedic Surgery School of Medicine Kitasato University Sagamihara Kanagawa Japan; ^2^ Department of Biomedical Engineering and Rehabilitation Kitasato University School of Allied Health Sciences Sagamihara Kanagawa Japan

**Keywords:** Arthrolysis, bone deformities, elbow contractures, mobilization, preoperative simulation, three‐dimensional full‐scale bone modeling

## Abstract

It is often difficult to treat for elbow contractures by malformation of bones. We planned a mobilization of elbow with using three‐dimensional full‐scale bone modeling. We found it was effective to use it in preoperative planning because we could recognize the elements of contractures in every deformity.

## Introduction

Contracture of the elbow joint typically results in a substantial loss of upper extremity function, which can jeopardize the ability to independently perform daily activities. Treatment of elbow contractures is therefore important to improve quality of life. Patients with a reduced range of motion (ROM) because of soft tissue abnormalities can achieve variable degrees of improvement with rehabilitation therapy, but surgery is the only treatment option for maximizing ROM, particularly in cases of contracture caused by bone deformity. However, predicting the postoperative ROM is often difficult because of anatomical differences in bone abnormalities among patients.

Orthopedic surgeons typically need to evaluate complex anatomical changes intraoperatively. Surgical treatment of patients with elbow contractures caused by bone deformity requires particular care, and the postoperative outcome is highly dependent on the experience and skill of the surgeon 1. Plain radiographs provide adequate information regarding the degree of deformity in the coronal and sagittal planes, but do not provide sufficient information about complex three‐dimensional (3D) deformities 2, 3.

Patterns of bone deformity vary among individual patients. To aid in the preoperative evaluation of bone anatomy and simulation of the planned surgery, we used current radiofrequency and computer technologies to obtain full‐scale 3D bone models based on computed tomography (CT) data. These 3D models have been used by surgeons at our institution to evaluate the complex anatomy of bone deformities when performing surgeries for scoliosis, femoral osteotomy, and periacetabular osteotomy, and good corrections of the deformities have been achieved. Here, we report the use of a 3D elbow model to plan surgery in three patients with severe elbow contractures due to bone deformities. Demographic data of patients in this case reports are shown below (Table 1).

**Table 1:** Demographic data of patients in this case series

Case No.	Sex	Age (years)	Injured side	Dominant side	Cause	Treatment before mobilization
1	Female	69	Right	Right	Open fracture‐dislocations	ORIF, Skin grafting
2	Male	30	Right	Right	Open fracture	ORIF, Skin grafting, Muscle flap surgery
3	Male	51	Left	Right	Osteoarthritis	None

## Case Reports

### Case 1

**Figure 1:**
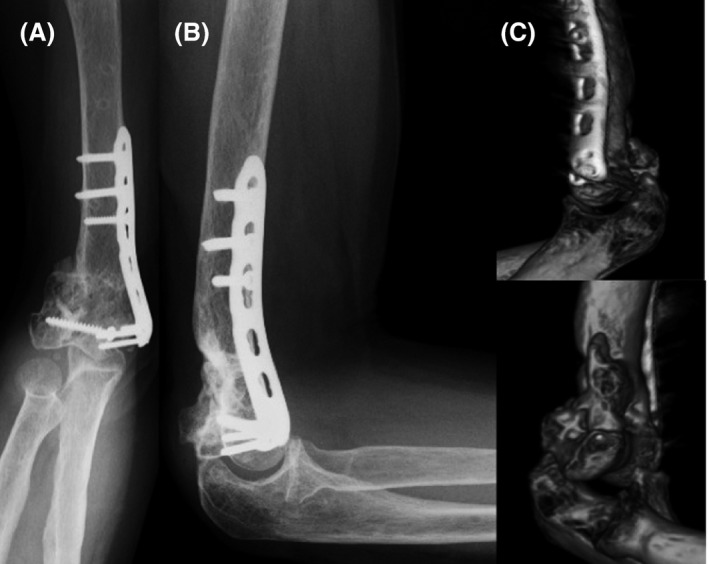
ORIF was performed after sustaining an open dislocation fracture (A), (B) and bone spurs are seen on CT (C).

**Figure 2:**
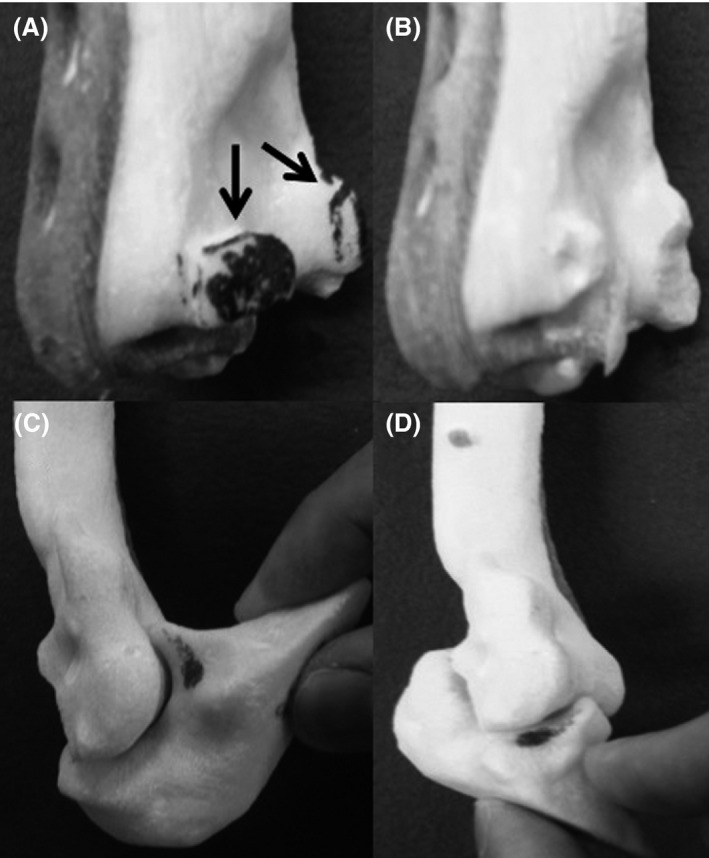
Bone spurs marked in black (→) (A) was cut (B) and simulation was performed. Estimated ROM was 135 degrees flexion (C) and −40 degrees extension (D).

A 69‐year‐old female sustained an open fracture‐dislocation of her right elbow in a car accident. She had undergone three previous surgical procedures, including open reduction and internal fixation (ORIF), and skin grafting, but had severely restricted elbow movement at 22 months after her last ORIF procedure, with 105° of flexion, −60° of extension, and 80° of both pronation and supination (Table 2). Radiography and CT showed the presence of multiple bone spurs that were causing elbow contracture (Fig. 1). Her contracture could have been treated by either total elbow arthroplasty (TEA) or surgical mobilization of the joint. Although TEA typically improves ROM and stabilizes the joint, in this case, there was obvious loss of bone because of the open fracture, and revision TEA would have been difficult to perform if a postoperative infection developed. In addition, the patient preferred not to undergo TEA. We therefore used a 3D model of her elbow to evaluate the joint and simulate possible surgical procedures (Fig. 2). We made markings at the point of bone resection in the model.

**Table 2:** Clinical outcome data treated by mobilization with using 3D model

Case No.	Follow‐up (months)	Operative time (min)	Flexion arc (°)				Extension arc (°)				MEPS	
			Pre	Simulation	Intra	Last visit	Pre	Simulation	Intra	Last visit	Pre	Post
1	41	112	105	135	140	135	−60	−40	−35	−35	75	90
2	36	210	125	130	130	130	−55	−30	−40	−35	55	80
3	29	295	110	130	130	120	−30	−20	−15	−20	75	80

Pre, preoperatively; Intra, intraoperatively; Post, postoperatively; MEPS, Mayo Elbow Performance Score. The Mayo Elbow Performance Score is classified as excellent, >90; good, 75–89; fair, 60–74; poor, <60.

**Figure 3:**
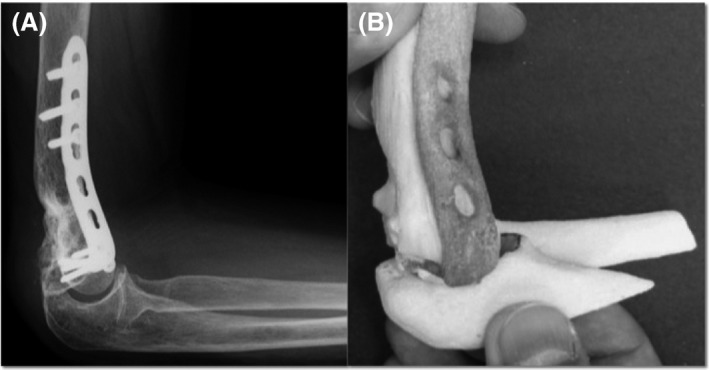
Osteotomy was performed (A) almost same as preoperative simulation (B).

The simulations indicated that resection of the bone spurs would result in an estimated 135° of flexion and −40° of extension and therefore decided to perform joint mobilization. The patient underwent open elbow arthrolysis via a combined medial‐lateral approach. The model was brought into the operating room, and the bone spurs were resected according to the markings we made as preoperative simulation, resulting in an intraoperative improvement of the ROM similar to that predicted based on the simulation, with 140° of flexion and −35° of extension (Fig. 3). The MEPS improved from 75 preoperatively to 90 postoperatively. Thirty months after surgery, her ROM improved to 135° of flexion, −35° of extension, 90° of pronation, and 90° of supination, and she was satisfied with the treatment outcome.

### Case 2

A 30‐year‐old man who had sustained an intra‐articular fracture of the right elbow in a car accident when he was an infant had undergone ORIF, skin grafting, and muscle flap surgery to treat the injury. However, as his elbow movement remained restricted, he consulted us regarding the possibility of surgical treatment. Upon examination, his right elbow showed 125° of flexion, −55° of extension, 70° of pronation, and 90° of supination.

**Figure 4:**
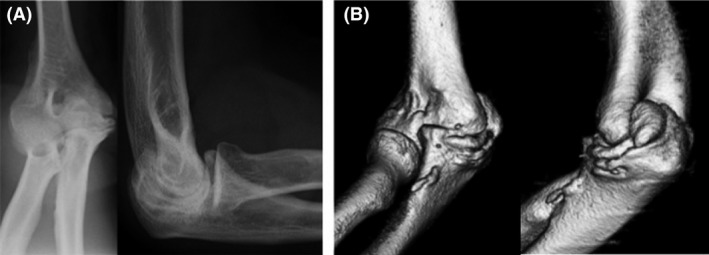
Bony protuberances are seen on the anteroposterior and lateral radiographs (A) and 3D CT (B).

**Figure 5:**
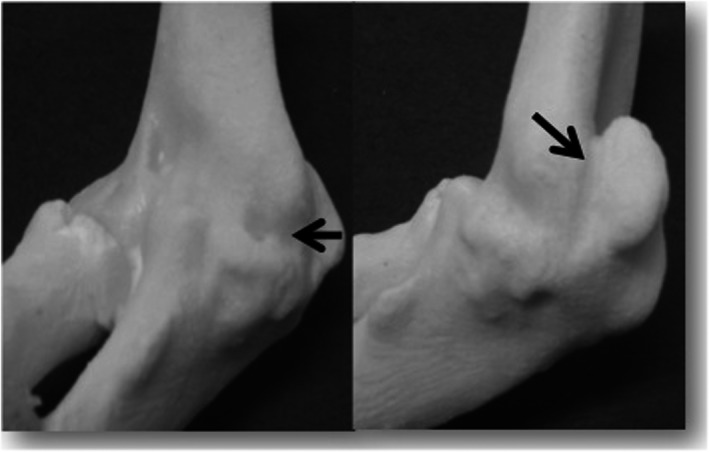
Preoperative simulation using the 3D model. Bony protuberances marked in black (→) were surgically resected.

**Figure 6:**
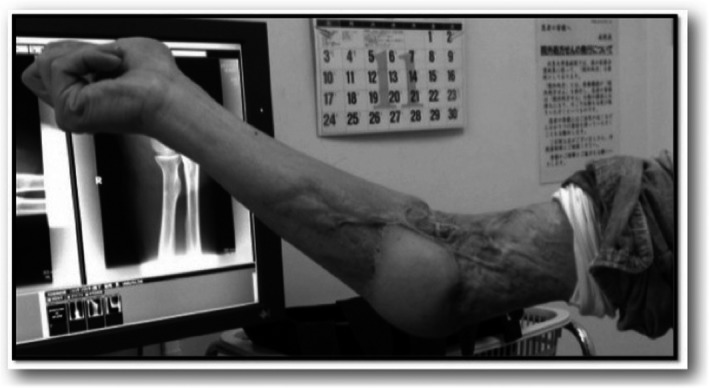
Skin grafting and muscle flap surgery was performed in childhood.

Radiography and CT showed malunion of the fracture and the presence of bony protuberances (Fig. 4). We performed open elbow arthrolysis via a combined medial‐lateral approach. Although the surgery was performed according to preoperative simulation using a 3D model (Fig. 5), the resulting intraoperative range of extension was less than expected because of skin contracture and muscle shortening resulting from his previous surgery (Fig. 6). The MEPS improved from 55 preoperatively to 80 postoperatively. Although postoperative physical therapy resulted in a slight improvement in the ROM, at the time of his last follow‐up, the patient had 135° of flexion and −35° of extension, and the same pronation and supination as before surgery.

### Case 3

A 51‐year‐old male begun to feel restricted ROM in his left elbow that was not attributable to trauma. Elbow contracture exacerbated gradually over a 10‐year period and he was diagnosed with osteoarthritis. Upon examination at our institution, his left elbow showed 110° of flexion, −30° of extension, 90° of pronation, and 90° of supination, and he desired to undergo surgery aimed at improving his ROM.

**Figure 7:**
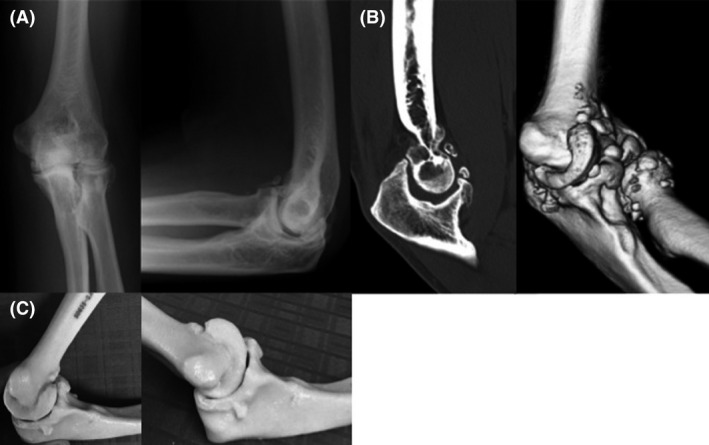
Radiography (A) and CT (B) showed the presence of bony protuberances. Estimated ROM was 130°of flexion and −20° of extension (C).

Radiography and CT showed the presence of bony protuberances (Fig. 7). We initially performed synovectomy by elbow arthroscopy and then performed open elbow mobilization via a combined medial‐lateral approach. The prominent osteophyte in the coronoid process and olecranon and bulges in both fossae were trimmed according to preoperative simulation using a 3D model (Fig. 7), and intraoperative ROM was 130° of flexion and −15° of extension. The patient had 120° of flexion and−20° of extension, and the same pronation and supination as before surgery at the time of his last follow‐up. The MEPS improved from 75 preoperatively to 80 postoperatively, and he was satisfied with the treatment outcome.

## Materials and Methods

We retrospectively reviewed three surgically treated patients (two men and one woman) with elbow contractures. Informed consent was obtained from each patient. Two patients had sustained an open fracture in a car accident, and the third patient had a contracture caused by elbow osteoarthritis. The mean age of patients at the time of surgery was 50 years (range, 30−69 years). The mean follow‐up period was 35.3 months (range, 29−41 months). None of the patients in this series had preoperative symptoms of ulnar neuropathy at the elbow. All of the procedures were performed by a single elbow surgeon (TK) and involved the right elbow in two cases, and the left elbow in one case. In all but one case, the surgically treated elbow was of the dominant arm. To assist in operative planning, a full‐scale 3D model of each patient's elbow was generated based on CT data. For the generation of the 3D models, 1‐mm‐thick slices were used, although a useful 3D model can be constructed with slices up to 5 mm in thickness. The CT data were sent to a company (Sony EMCS Co., Aichi, Japan) that manufactured the models using a blended salt called “modecis” (80% sodium chloride, 5% polyvinyl alcohol, and 15% magnesium sulfate 15%; Tomita Pharmaceutical Co., Ltd., Tokushima, Japan). The models could be cut and reconstructed repeatedly using a bonding agent, thereby enabling the separate evaluation of each bone, in addition to flexion, extension, pronation, and supination.

During surgery, bony protuberances were excised and as little cartilage as possible was removed from the trochlea and capitellum. The generated 3D elbow models allowed us to perform preoperative simulations and to make decisions concerning the amount of the bony protuberances to be removed. The models were brought into the operating room and referred to during the surgical procedures.

The surgeries were performed with two patients in supine positions and one patient in the prone position. An air tourniquet was used in all patients. The ulnar nerve was released in all patients, but was not transposed anteriorly. Two weeks postoperatively, the patients were fitted with an arm sling. A single operator (KT) measured the ROM using a goniometer. Preoperative, intraoperative, and postoperative ROM and the Mayo Elbow Performance Score (MEPS) were used as outcome measures.

## Results

Demographic and clinical outcome data of the three patients are shown in Tables 1 and 2, respectively. The average preoperative flexion and extension were 113.3° (range, 105°−125°) and −48.3° (range, −60° to −30°), respectively. The average flexion and extension simulated by the 3D models were 131.7° (range, 130°−135°) and −30.0° (range, −40° to −20°), respectively. The average intraoperative flexion and extension were 133.3° (range, 130°−140°) and −30.0° (range, −40° to −15°), respectively. Average flexion and extension at the time of the final follow‐up averaged 128.3° (range, 120°−135°) and −30° (range, −35° to −20°). The mean MEPS improved from 68.3(range, 55−75) to 83.3 (range, 80−90). None of the patients had valgus or varus instability after mobilization surgery.

## Discussion

Most activities of daily living can be performed with 100° of elbow flexion (30° to 130°) and 100° of forearm rotation (50° of pronation and 50° of supination) 4. Therefore, the aim of elbow joint mobilization surgery is to achieve a ROM required for this functional arc. However, in patients with bone deformities, preoperative estimation of postoperative ROM is often difficult, particularly in cases with complex abnormalities. Preoperative surgical simulation using a full‐scale 3D model was reported to be useful for improving the accuracy and safety of surgery in cases of severe anatomical deformity 5, 6, 7, as such models allow for anatomical evaluation and intraoperative confirmation of osteotomy sites. Three‐dimensional models can also be used to estimate the risk of intraoperative fracture or instability resulting from excessive bone excision and can be repeatedly cut and reconstructed using a bonding agent, thereby providing useful preoperative information. Moreover, 3D models can be brought into the operating room, allowing for intraoperative referral.

Elbow contractures may result from both extrinsic and intrinsic factors 8, 9, 10. Extrinsic factors typically involve only the soft tissues, such as the capsules, ligaments, and muscles, or a combination of these structures 8, whereas intrinsic factors are mainly associated with the degeneration of articular cartilage and typically require treatment by TEA or interposition arthroplasty 8, 9, 11. In the present study, because all patients had intrinsic contractures in their elbow, 3D models were necessary. In all cases, the postoperative ROM that was estimated from simulations with the generated 3D elbow models was the same as, or less than, the ROM required for a functional elbow. In patients who also have skin contractures, as in Case 2, it is difficult to determine the optimal site for osteotomy based only on intraoperative findings, because the elbow cannot be moved to the targeted angle, even after performing the osteotomy. This difficulty tends to prolong the operation time, which increases the risk of infection 12, and means that the surgical outcome is largely influenced by the experience and skill of the surgeon. In the present cases, the elbow anatomy was evaluated preoperatively using the 3D models, and the surgical procedure was performed according to the preoperative plan that was developed with the aid of the 3D model.

If accurate osteotomy through the aid of a 3D model is achieved, extrinsic factors, such as capsules, ligaments, and muscles, would also be expected to improve after postoperative rehabilitation. For all procedures that were performed following preoperative simulation using the full‐scale 3D elbow models, the positions of bone sections and target ROM were decided before surgery. The precise selection of the site for osteotomy is also possible using 3D models 9.

Although all patients had severe bone deformities, the full‐scale 3D elbow models enabled accurate evaluation of all bones. This technique may therefore be useful for the evaluation and treatment of almost all elbow deformities. As none of the patients had severe limitations of pronation or supination, or axial malalignment of the elbow joints, corrective osteotomy was not required. If osteotomies had been necessary, these procedures could also have been planned using 3D models that included the entire forearm. Notably, however, because the flexion axis can change during elbow motion, particularly in patients with articular deformities, it may be difficult to predict elbow motion based only on our 3D modeling approach, as these models lacked cartilage and ligaments. Other disadvantages of this method include the increased exposure of the patient to radiation during CT scanning and the relatively high cost of producing the 3D model. However, as the preoperative simulations greatly improved the accuracy and safety of the surgical procedures, which had excellent clinical outcomes, the risks associated with radiation exposure may be outweighed by the treatment benefits 3. The 3D models used in the study cost more than 100,000 Japanese yen (≈ 1000 US dollars) each and may therefore be cost prohibitive for widespread use. Although the 3D models described here are not commercially available at large scale, the continued development of 3D printers may allow for such models to be constructed on site at low cost in the near future 13.

## Conclusions

All of the patients in this series had significant improvements in the ROM of the elbow at the time of the final follow‐up compared with the preoperative ROM. Preoperative simulations using full‐scale 3D models provide useful and accurate information on the anatomical data of complicated bone deformities.

## Disclaimer

The authors, their immediate families, and any research foundations with which they are affiliated have not received any financial payments or other benefits from any commercial entity related to the subject of this article.

## Conflict of Interest

None declared.

## Authorship

TK: for experimental design. KO: for experimental design. KF: for acquisition of data. HF: for acquisition of data. NT: for analysis and interpretation of data. MT: for drafting the manuscript. Those people or groups who provided support to the publication but not as a direct contributor may be included in the acknowledgement section of the publication.
